# Comparative Genomics and In Vitro Plant Growth Promotion and Biocontrol Traits of Lactic Acid Bacteria from the Wheat Rhizosphere

**DOI:** 10.3390/microorganisms9010078

**Published:** 2020-12-30

**Authors:** Sabrina Strafella, David J. Simpson, Mohammad Yaghoubi Khanghahi, Maria De Angelis, Michael Gänzle, Fabio Minervini, Carmine Crecchio

**Affiliations:** 1 Department of Soil, Plant and Food Sciences, University of Bari Aldo Moro, Via Amendola 165/a, 70126 Bari, Italy; sabrina.strafella@uniba.it (S.S.); mohammad.yaghoubikhanghahi@uniba.it (M.Y.K.); maria.deangelis@uniba.it (M.D.A.); fabio.minervini@uniba.it (F.M.); 2 Department of Agricultural, Food and Nutritional Science, 410 Agriculture/Forestry Centre, University of Alberta, Edmonton, AB T6G 2P5, Canada; djsimpso@ualberta.ca (D.J.S.); mgaenzle@ualberta.ca (M.G.)

**Keywords:** plant growth promoting bacteria, lactic acid bacteria, wheat rhizospheric soil, comparative genomic analysis, bacteriocins, 3-indolacetic acid production, potassium solubilization, phosphate solubilization, antifungal activity, antibacterial activity

## Abstract

This study aimed to isolate lactic acid bacteria (LAB) from wheat rhizosphere, to characterize their in vitro plant growth promoting activities and to differentiate plant-associated LAB from those associated with foods or human disease through comparative genomic analysis. *Lactococcus lactis* subsp. *lactis* and *Enterococcus faecium* were isolated using de Man-Rogosa-Sharpe (MRS) and Glucose Yeast Peptone (GYP) as enrichment culture media. Comparative genomic analyses showed that plant-associated LAB strains were enriched in genes coding for bacteriocin production when compared to strains from other ecosystems. Isolates of *L. lactis* and *E. faecium* did not produce physiologically relevant concentrations of the phyto-hormone indolacetic acid. All isolates solubilized high amount of phosphate and 12 of 16 strains solubilized potassium. *E. faecium* LB5, *L. lactis* LB6, LB7, and LB9 inhibited the plant pathogenic *Fusarium graminearum* to the same extent as two strains of *Bacillus* sp. However, the antifungal activity of the abovementioned LAB strains depended on the medium of cultivation and a low pH while antifungal activity of *Bacillus* spp. was independent of the growth medium and likely relates to antifungal lipopeptides. This study showed the potential of rhizospheric LAB for future application as biofertilizers in agriculture.

## 1. Introduction

Agriculture is an important economic sector in many countries and, according to FAO, 37% of the global land area is dedicated to agriculture [1]. Conventional agriculture produces high yields and seems to be the most appropriate solution to the estimated increase of the world population [2]. However, it relies on the use of chemical fertilizers, which are responsible for water and soil pollution, soil degradation and biodiversity loss [3]. Conventional agriculture also employs pesticides to control phytopathogens that cause the loss of an estimated 9–16% of important cereal crops such as wheat, rice, and maize [4].

Organic agriculture excludes use of synthetic chemical fertilizers, pesticides, and herbicides [5] and improves soil fertility through the incorporation of legumes and compost, determining an increase of specie biodiversity in the whole ecosystem [6]. Organic agriculture produces 13–34 % lower yields when compared to conventional cropping systems [7]. Eco-friendly and efficient solutions for pest control in organic agriculture may complement or substitute the use of pesticides in conventional agriculture.

The plant rhizosphere is rich in nutrients released by root exudates that create a suitable niche for proliferation of microorganisms. Microorganisms reach high cell densities and the rhizosphere is a complex ecosystem [8]. Organisms in the rhizosphere include bacteria, fungi, nematodes, algae, protozoa, arthropods, and archaea but bacteria are the most abundant (10^8^–10^9^ cells·g^−1^ of rhizospheric soil) [9]. Among bacteria, Gram-negative bacteria predominate [10]. Not all microorganisms populating the rhizosphere, termed “rhizobacteria”, have been characterized because it is not easy to culture them [11]. Many studies suggest that only 1–5% of total rhizobacteria are cultivable and abiotic stress could halve that number [8,9]. Some rhizobacteria improve plant growth and productivity through various mechanisms of action. Correspondingly, rhizobacteria are commercially used as plant growth promoting rhizobacteria (PGPR) or biofertilizers [12]. Depending on the basis of the mechanisms of action, PGPR are considered as biostimulants when they improve nitrogen fixation, phosphate and potassium solubilization, or produce phytohormones, or biocontrol agents when they produce antimicrobial compounds with activity against phytopathogens [13,14,15]. Introduction of PGPR in conventional agriculture is a strategy to reduce its environmental impact without decreasing plant productivity. The predominant genera used as PGPR are *Bacillus* and *Pseudomonas* [16]. Metagenomics analyses of plant microbiomes, including rhizobacteria, identified additional organisms including lactic acid bacteria (LAB); which are, however, hardly detectable because of their low abundance in the plant-soil ecosystem [17,18,19].

LAB are microaerophilic Gram-positive bacteria which play an important role in food fermentations but also include commensals of vertebrates and insects as well as pathogens [20]. Food fermenting LAB, particularly species grouped in the *Lactobacillaceae*, have a safe tradition of use in food [21], but this group also includes pathogens or opportunistic pathogens. For instance, *Enterococcus faecium* has evolved as vertebrate commensals and occur in food fermentations but specific lineages of *E. faecium* also are a leading cause of hospital-acquired infections with multi-drug resistant bacteria [22,23]. Strains of *E. faecium* which lack the virulence genes typical of hospital isolates have been approved as feed additives [24]. LAB also include plant-associated organisms, although their abundance and diversity in the rhizosphere is lower when compared to other bacteria [20]. Although they do not have a reputation of having as useful properties as bacilli and *Pseudomonas* species, some species of LAB were assessed with regards to their plant growth promoting activity and were reported to increase yield, improve seed germination [25], and the chlorophyll [26] and amino acid content [27] of plants. Some reports also suggested a role of LAB as biocontrol agents against soil-borne phytopathogens [25,28]. Nevertheless, the potential of LAB as PGPR is still under-explored. Therefore, the aims of this study were: (i) to isolate LAB from the rhizospheric soil collected from two cultivars of durum wheat during different growth stages; (ii) to determine whether any of the useful properties of the established PGPB are also found in LAB, by characterizing their plant growth promotion and protection activities in vitro; (iii) to evaluate whether the plant associated LAB differ from strains of the same species that are associated with foods and/or with humans.

## 2. Materials and Methods

### 2.1. Samples Collection

Two varieties of durum wheat (*Triticum turgidum* subsp. *durum*), Senatore Cappelli and MARAKAS, were grown with conventional farming systems at two sites in Southern Italy, Turi (40°91′ N, 17°04′ E) and in Gravina (40°49′ N, 16°18′ E). Turi soil (pH 7.95, electric conductivity (EC) 0.57 dS·m^−1^, soil organic carbon (SOC) 1.69%, soil organic matter (SOM) 2.91%) and Gravina in Puglia soil (pH 7.63, EC 0.61 dS·m^−1^, SOC 1.26%, SOM 2.17%) were characterized by a clay loam texture. Wheat rhizospheric soil samples were collected during four different growth stages (tillering, elongation, earing, and physiological maturity) between January and June, months during which the average monthly temperature ranges from 4 to 30 °C. For each field and growth stage, ten soil samples were randomly collected, for a total of 80 samples, using the following protocol: (i) eradicating manually to a depth of 0–20 cm and shaking plants to remove soil in excess; (ii) storage of plant’s roots individually in sterile bags and transport to laboratory at 4 °C; (iii) collection of soil attached to the roots using a sterile spatula; (iv) sieving (0.5 mm). Then, the soil samples were randomly pooled to form two sub-samples considered replicas, each one obtained from five plants.

### 2.2. Isolation of Lactic Acid Bacteria from Wheat Rhizospheric Soil

Lactic acid bacteria (LAB) were isolated from wheat rhizospheric soil upon enrichment, as described by Chen et al. [18]. One gram of rhizospheric wheat soil of each sub-sample was added to 5 mL of de Man, Rogosa, and Sharpe (MRS, pH 6.2) or Glucose Yeast Peptone (GYP, pH 7) broth, both supplemented with cycloheximide (0.1 g·L^−1^). MRS was purchased from Oxoid Ltd. (Basingstoke, UK), except when used for culturing bacteria before antifungal assay. GYP was prepared in laboratory and contained 20.0 g·L^−1^ glucose, 10.0 g·L^−1^ yeast extract, 10.0 g·L^−1^ peptone, 10.0 g·L^−1^ sodium acetate, 5 mL salt solution (40.0 g·L^−1^ MgSO_4_·7H_2_O, 1.6 g·L^−1^ MnSO_4_·4H_2_O, 2.0 g·L^−1^ FeSO_4_·7H_2_O, 2.0 g·L^−1^ NaCl) [18].

After 24 h of incubation at 30 °C, 1 mL of enrichment broth was diluted with Ringer solution 25% composed of 0.225 % NaCl *w*/*v*, 0.0105% KCl *w/v*, 0.0045% CaCl_2_ *w/v*, 0.005% NaHCO_3_ *w/v*, and 0.0034% citric acid *w/v*, and inoculated by pour-plate method on MRS and GYP agar plates. After incubation at 30 °C for 48 h, a number of colonies equal to the square root of the total number observed in plates coming from the highest dilution was randomly picked up. Gram-positive, catalase-negative, non-motile rod- and coccus-shaped LAB were cultivated in MRS or GYP at 30 °C for 24 h and re-streaked onto the same agar media. All isolates considered for further analyses were able to acidify the culture medium.

### 2.3. Molecular Biotyping and Identification of LAB Isolates

The isolates were typed by randomly amplified polymorphic DNA (RAPD)-PCR. Colony PCR was performed upon thermal shock of each colony and, subsequently, inserting the colony into the following reaction mix: 1× Reaction Buffer (EuroClone, Pero, MI, Italy), 2.5 mM MgCl_2_ (EuroClone), 0.2 mM each dNTP (EuroClone), 1 µM M13 primer (5′-GAGGGTGGCGGTTCT-3′) (Sigma-Aldrich, St. Louis, MO, USA) [29], 80 ng µL^−1^ bovine serum albumin (Sigma-Aldrich), 3 U of Taq DNA polymerase (EuroTaq, EuroClone). The amplification reactions were carried out using MyCycler™ thermal cycler (Bio-Rad Laboratories Inc., Hercules, CA, USA) and following the conditions described by Zapparoli et al. [30] with some modifications: 95 °C for 10 min, 35 cycles of 95 °C for 1 min, 45 °C for 30 s, 72 °C for 2 min, and a final extension of 72 °C for 5 min. The PCR products (3 μL) were separated by electrophoresis at 100 V for 60 min on a 1% (*w/v*) agarose gel, and the electrophoretic profile was detected by Bio-Rad Gel Doc 2000 System image detector and processed using the Image Lab software.

Identification of LAB was carried out upon partial sequencing of 16S rRNA gene (V3-V4 regions), using the F357 forward and R1401 reverse primers [31]. The reaction mix contained 1× Wonder Taq Reaction Buffer (EuroClone), 50 pmol of each primer (Eurofins Genomics, Ebersberg, Germany), 80 ng·µL^−1^ bovine serum albumin, 3 U of Taq DNA polymerase. The PCR conditions were: 95 °C for 10 min, 35 cycles of 95 °C for 30 s, 63 °C for 30 s, 72 °C for 2 min and a final extension of 72 °C for 7 min. PCR amplicons of 1044 bp were purified using Wizard SV Gel and PCR Clean-Up System kit (Promega) following the manufacturer’s instructions. After quantification of amplicons through the spectrophotometer NanoDrop ND-1000, PCR products were sequenced by Eurofins Genomic.

### 2.4. In Vitro Characterization of Lactic Acid Bacteria

#### 2.4.1. Quantification of 3-Indolacetic Acid

The strains were incubated in MRS and GYP broth, enriched by 0.2% L-Tryptophan, for 72 h at 28 °C. After incubation, the bacterial suspension was centrifuged at 5500× *g* for 15 min and 1 mL of cell-free supernatant was added to 4 mL Salkoswki’s reagent prepared as described by Gordon and Weber [32]: 36% H_2_SO_4_ solution containing 6.2 mM FeCl_3_. The mix was left in the dark at room temperature for 20 min, then the absorbance at 535 nm was read using a spectrophotometer (Ultrospec 4000 UV–visible Spectrophotometer, Pharmacia Biotech). The calibration curve was constructed using pure 3-indolacetic acid (Sigma-Aldrich) as standard at different concentration in the range of 2–30 mg·L^−1^.

#### 2.4.2. Phosphate Solubilization and Phosphorus Quantification

A screening on NBRIP (National Botanical Research Institute’s Phosphate) agar (1% *w/v* glucose; 0.5% *w/v* Ca_3_(PO_4_)_2_; 0.5% *w/v* MgCl_2_·6H_2_O; 0.025% *w/v* MgSO_4_·7H_2_O; 0.02% *w/v* KCl; 0.01% *w/v* (NH_4_)_2_SO_4_; 1.5% *w/v* agar, pH 7) was performed to assay the ability of the strains to solubilize phosphate [33]. 10 µL of bacterial suspension, grown for 24 h in GYP or MRS, were spread in triplicates onto NBRIP agar plates and incubated for 15 days at 30 °C. The diameter of a halo zone around the colony was measured to calculate the Phosphate Solubilization Index (PSI).

After screening on NBRIP agar, quantification of solubilized phosphorous was carried out, following the colorimetric method of Watanabe and Olsen [34]. 100 µL of cell-free supernatant, harvested after 7 days of incubation in NBRIP broth at 28 °C, were added to 4 mL of a solution obtained dissolving 1.056 g of ascorbic acid in 200 mL 5N H_2_SO_4_ solution also containing 6.2 mg·mL^−1^ of ammonium molybdate and 0.14 mg·mL^−1^ of antimony potassium tartrate. Double distilled water was added until 100 mL of final volume and after 10 min of incubation at room temperature, the absorbance was read at 650 nm. The calibration curve was constructed using ascorbic acid at different concentration in the range 0–0.6 µg·mL^−1^.

#### 2.4.3. Potassium Solubilization

Aleksandrov agar medium was used to assay the strains’ ability to solubilize potassium. The medium contained 3.5 g·L^−1^ glucose, 0.5 g·L^−1^ MgSO_4_·7 H_2_O, 0.1 g·L^−1^ CaCO_3_, 0.0005 g·L^−1^ FeCl_3_, 2 g·L^−1^ Ca_3_PO_4_, 12 g·L^−1^ agar and 1.0 g·L^−1^ mica powder, pH 7 [35]. Ten µL of bacterial suspension were spotted in triplicate on Aleksandrov agar and, after 4 days of incubation at 28 °C, the halo zone around the colony was measured to calculate solubilization index of potassium (KSI).

After screening on Aleksandrov agar, solubilized potassium was quantified, according to Yaghoubi Khanghahi et al. [36]. In detail, 100 µL of each bacterial suspension cultured for 24 h were inoculated, in triplicate, in 10 mL of Aleksandrov broth. After incubation (14 days at 28 °C), bacterial suspensions were centrifuged (4032× *g*, 20 min, 4 °C) and the supernatant was 0.2 μm filtered. Then, the supernatants were diluted 1:5 in double distilled water and 10 µL of a 100 mg·L^−1^ Gallium solution as internal standard were added. 10 μL of the abovementioned solution were spread onto a clean siliconized quartz disk and left to dry on a hot plate (SD300, Stuart) to form a circular thin film ready for analysis [37]. Quantitative analysis of potassium was carried out by total-reflection X-ray fluorescence spectrometry (TXRF), using an S2Picofox TXRF Spectrometer (Bruker Nano GmbH, Berlin, Germany) and spectra were analyzed by SPECTRA 7 software (Bruker Nano GmbH, Berlin, Germany). The intensity of the fluorescence emitted by the samples was measured, processed by referring to the internal standard, and converted in concentration of solubilized potassium [37].

#### 2.4.4. Antifungal Activity

*Aspergillus niger* FUA5001, *Penicillium roqueforti* FUA5004, and *Fusarium graminearum* G-1, belonging to the Culture Collection of the University of Alberta (Edmonton, Canada), were used to test antifungal activity of LAB. *Bacillus velezensis* FUA2155 and *Bacillus amyloliquefaciens* Fad 82 [38], routinely cultured in Luria–Bertani (LB) broth, were used as positive controls because of the widespread ability of plant-associated bacilli to produce diverse antifungal lipopeptides [39]. Fungi were grown on Potato Dextrose Agar (PDA) at 25 °C in the dark for 7 days and then the spores were collected in sterile physiological solution. The suspension was filtered, and the number of the spores was counted using the hemocytometer (Fein-Optik, Jena, Germany). The suspension was first diluted until 1 × 10^4^ spore/mL and then further diluted 1:30. Before the assay, bacterial strains were cultured in broths lacking sodium acetate: either GYP or laboratory-made MRS (mMRS: 10 g·L^−1^ peptone, 8 g·L^−1^ ‘Lab-Lemco’ powder, 4 g·L^−1^ yeast extract, 20 g·L^−1^ glucose, 1 mL·L^−1^ sorbitan mono-oleate, 2 g·L^−1^ dipotassium hydrogen phosphate, 2 g·L^−1^ triammonium citrate, 0.2 g·L^−1^ magnesium sulphate heptahydrate, 0.05 g·L^−1^ manganese sulphate tetrahydrate). The screening was performed in triplicate using 96-well plates. Each well contained 100 µL of culture medium routinely used for the bacteria tested, but lacking sodium acetate, and 15 µL of bacterial suspension cultured for 24 h. The plates were incubated at 30 °C for two days until bacterial growth; after that, 15 µL of a spore suspension were added. The 96-well plates were incubated at 25 °C, and day by day the mold growth was monitored against three controls consisting of only culture media (acidified to pH 4.5 using lactic acid) and spore suspension [40]. Antifungal activity was expressed as mean of the mold-free days and represented as a heatmap using R software.

#### 2.4.5. Antibacterial Activity

*Pantoea agglomerans* NCPPB:2519, *Pectobacterium rhapontici* NCPPB:1578, *Pseudomonas syringae* NCPPB:281, *Pectobacterium carotovorum* subsp. *carotovorum* NCPPB:312, belonging to the National Collection of Plant Pathogenic Bacteria (Fera Science Ltd., London, UK), and *Pseudomonas fulva*, belonging to the Culture Collection of the University of Alberta, were used as indicators to test antibacterial activity of LAB. *P. fulva* was cultured in tryptic soy broth (TSB) at 37 °C for 24 h, whereas the other bacteria were cultured in nutrient broth (NB) at 25 °C for 24 h under shaking.

The screening was carried out in triplicate using the minimum inhibitory concentration (MIC) protocol [41]. In 96-well plates, 100 µL of culture medium of each phytopathogenic bacterial strain were added to each well. Then, 100 µL of cell-free supernatant of LAB collected from 24 h cultures were added in the wells of one column and diluted by serial two-fold dilutions. The plates were then inoculated with 50 µL of a cell suspension of the indicator strains that was diluted to 1–5 × 10^6^ cfu/mL; one column remained uninoculated and served as negative control; one column was inoculated but contained no culture supernatant and served as positive control. Growth was assessed by measuring the optical density at 600 nm after 24 h of incubation (Biolog Inc., Hayward, CA, USA). A second test was performed as described above, with the only change of using the cell-free supernatant of LAB after pH adjustment to 7 with 5 M NaOH. pH values of suspension contained in each well were measured using a HI1083P electrode (Hanna Instruments Italia, Ronchi di Villafranca Padovana, Italy).

The MIC, representing the lowest dilution of supernatant able to totally inhibit phytopathogenic bacteria, was expressed as the ratio of the supernatant volume to the total volume (%, *v/v*).

### 2.5. Comparative Genomic Analysis

Genomes of two strains of *Lactococcus lactis* and nine strains of *Enterococcus faecium* were sequenced by paired end sequencing on a HiSeq2500 platform with 50-fold coverage by service of the Génome Québec Innovation Centre (Montreal, QC, Canada), followed by quality control with the FastQC tool, assembly using SPAdes and MeDuSa, and annotation on the rapid annotations using subsystems technology (RAST) server as described [42]. Genomic metadata for *E. faecium* and *L. lactis* was downloaded using NCBImeta [43]. From this data, 102 *E. faecium* strains were selected. Clinical strains (38) were rarified by selecting one strain from each depositing source. Plant strains were selected by selecting all NCBI strains from plant, soil or fresh water sources (35). The nine strains isolated in this study were included in the plant group (44). All available strains isolated from healthy non-domestic animals were included in the animal group (29). *L. lactis* strains (77) were split into dairy and plant groups. The 47 dairy strains were selected from isolates from backslopped dairy cultures, whereas the plant group consisted of all available strains isolated from plants (28) and two of the strains isolated in this study.

Comparative genomics was carried out after annotating the genomes with Prokka [44] and creating gene presence/absence table and core genome alignments with Roary [45]. Analysis of the enriched for genes in the different groups was carried out using Scoary [46]. The phylogenetic trees were generated from the core genome alignments using FastTree 2.1 [47] and the trees were visualized using ITOL [48]. *Enterococcus faecalis* V583 (GCF_000407305.1) and *Lactococcus garvieae* ATCC 49156 (GCF_000269925.1) were used as outgroups.

### 2.6. Statistical Analysis

Statistical analysis was performed using SigmaPlot 14.0. All assays were conducted in triplicate biological repeats. One-way analysis of variance (ANOVA) with LSD post hoc analysis were performed with SigmaPlot to determine significant differences at a 5% probability level.

## 3. Results

### 3.1. Isolation and Identification of Lactic Acid Bacteria from Wheat Rhizospheric Soil

One hundred and fifty-one bacteria were isolated from durum wheat rhizospheric soil during tillering (10 isolates), elongation (55), earing (71), and physiological maturity (15). Seventy-two bacteria were isolated using GYP medium and the other 79 through MRS medium, two standard media for isolation of lactic acid bacteria. Seven isolates were catalase-positive and discarded, all other isolates were Gram-positive, catalase-negative, acidifying cocci. Upon RAPD-typing, 18 different electrophoretic profiles were observed. One representative strains of each RAPD-profile was characterized by partial sequencing of 16S rRNA genes. Nine strains belonged to *Lactococcus lactis* subsp. *lactis* and the remaining nine to *Enterococcus faecium* (Table 1).

### 3.2. Indolacetic Acid Production

**Figure 1:**
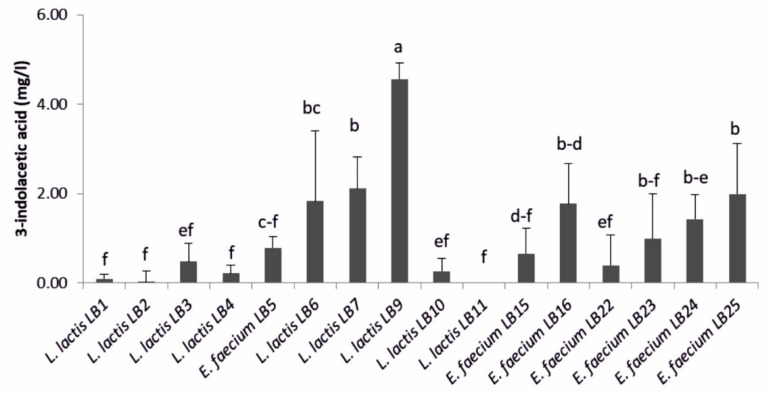
3-indolacetic acid amount produced by lactic acid bacteria (*n* = 3). Means (±SD) followed by the similar letter(s) are not significantly different at the 5% probability level (LSD test).

*L. lactis* subsp. *lactis* LB9 was the higher producer of 3-indolacetic acid (IAA) (4.6 ± 0.4 mg·L^−1^) compared to others LAB (Figure 1). Lower values were found for *L. lactis* subsp. *lactis* LB6, LB7 and *E. faecium* LB16, LB23, LB24, and LB25. Values close to zero were obtained for the other strains.

### 3.3. Phosphate Solubilization and Phosphorus Quantification

**Figure 2:**
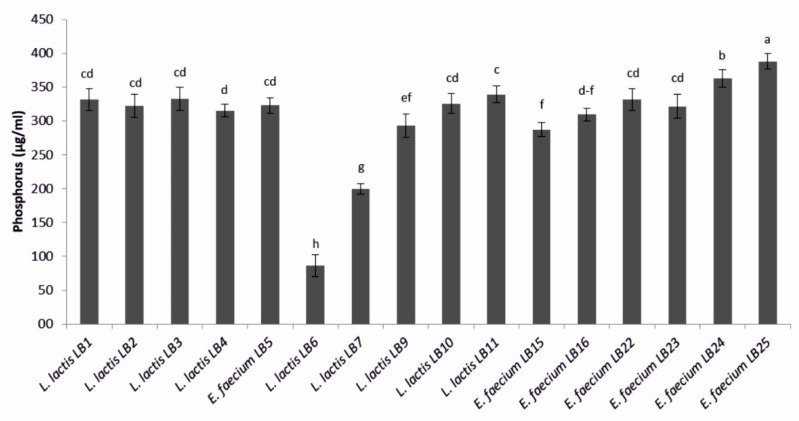
Amount of phosphorus solubilized by lactic acid bacteria. Means (±SD) followed by the similar letter(s) are not significantly different at the 5% probability level (LSD test).

All the LAB produced a phosphate solubilization halo, with PSI values ranging from 1.02 ± 0.03 to 1.87 ± 0.11 mm (Table 2). Most LAB were high phosphorous solubilizers, with values ranging from 200.0 ± 8.0 (*L. lactis* subsp. *lactis* LB7) to 388.2 ± 11.2 µg·mL^−1^ (*E. faecium* LB25). Only *L. lactis* subsp. *lactis* LB6 solubilized a lower amount of phosphorus (86.7 ± 16.0 µg·mL^−1^) (Figure 2).

### 3.4. Potassium Solubilization

Twelve LAB solubilized potassium from mica powder. *E. faecium* LB15, LB16, LB23, LB24, and LB25 produced a halo zone whose diameter was greater than 2 mm (Table 3). For the other seven potassium solubilizing bacteria, potassium solubilization index (KSI) ranged from 1.7 ± 0.1 to 2.0 ± 0.3 mm.

**Figure 3:**
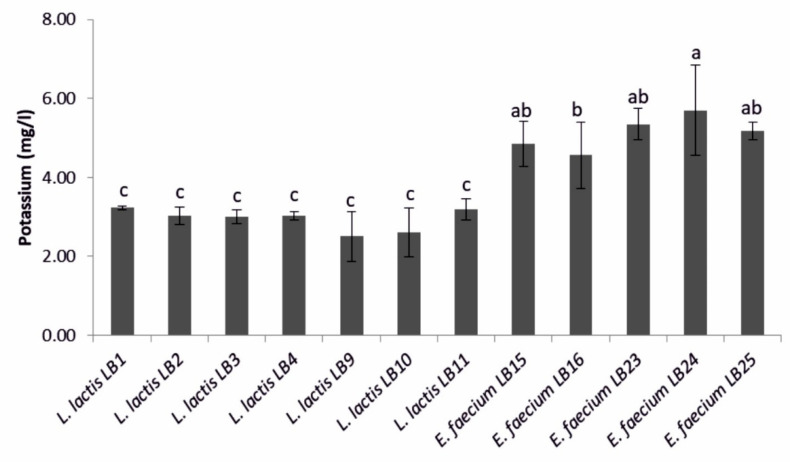
Amount of potassium solubilized by the lactic acid bacteria strains that showed a halo zone of potassium solubilization. Means (±SD) followed by the similar letter(s) are not significantly different at the 5% probability level (LSD test).

Among LAB showing a halo zone of potassium solubilization, strains of *E. faecium* were higher potassium solubilizers (4.6–5.7 mg·L^−1^) than *L. lactis* subsp. *lactis* (Figure 3). Among the *L. lactis* subsp. *lactis* strains tested, LB1 reached the highest value (3.2 ± 0.1 mg·L^−1^).

### 3.5. Antifungal Activity

**Figure 4:**
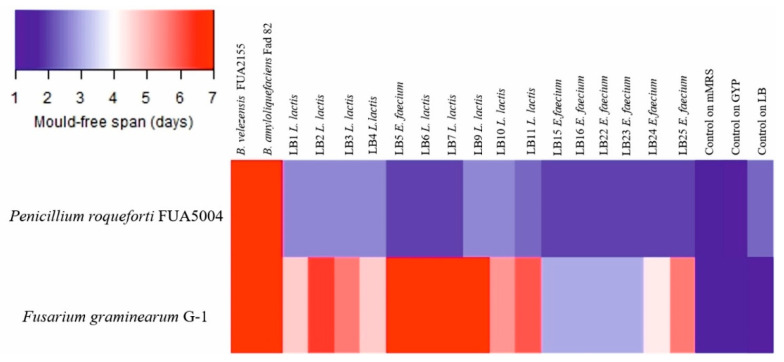
Heatmap representing the average of the mold-free days (*n* = 3). Controls on mMRS, GYP, and LB broths refer to wells containing just broth acidified to pH 4.5 and fungal spore suspension. *Bacillus velezensis* FU2155 and *Bacillus amyloliquefaciens* Fad82 were cultured in LB broth. LAB were cultured keeping the same culture medium used for the isolation step: *Lactococcus lactis* and *Enterococcus faecium* LB5 were cultured in mMRS broth; the remaining *E. faecium* were cultured in GYP broth.

*Bacillus velezensis* FUA2155 and *Bacillus amyloliquefaciens* Fad 82 showed the strongest antifungal activity (Figure 4). *E. faecium* LB5 and *L. lactis* subsp. *lactis* LB6, LB7, LB9 exhibited inhibitory activity against *Fusarium graminearum* that was comparable the bacilli (Figure 4); however, activity of *L. lactis* culture supernatants against *F. graminearum* was observed only when cultured in mMRS but not when cultured in GYP. *L. lactis* subsp. *lactis* LB1, LB2, LB3, LB4, LB10, LB11 and *E. faecium* LB24, LB25 had a lower activity against *F. graminearum* when compared to *Bacillus* sp. strains. None of the LAB inhibited germination of *A. niger* spores, whereas *Bacillus* sp. strains were inhibitory to *A. niger*. All the LAB strains showed only weak inhibitory activity against *P. roqueforti* when compared to the two *Bacillus* sp. strains (Figure 4).

### 3.6. Antibacterial Activity

Neutralized culture supernatant of any of the strains of *L. lactis* and *E. faecium* showed no inhibitory activity against the tested phytopathogenic bacteria. The cell-free supernatant of seven strains of *L. lactis* was weakly inhibitory against the *Pantoea*, *Pseudomonas*, and *Pectobacterium* species if the pH remained unadjusted, with MIC values ranging from 2 to 17% (Table S1). Supernatant of *E. faecium* was not inhibitory against phytopathogenic bacteria even if the pH of the culture supernatant remained unadjusted.

### 3.7. Comparative Genomic Analysis between Plant Associated Lactococci and Enterococci and Strains Dairy or Clinical Isolates of the Same Species

**Figure 5:**
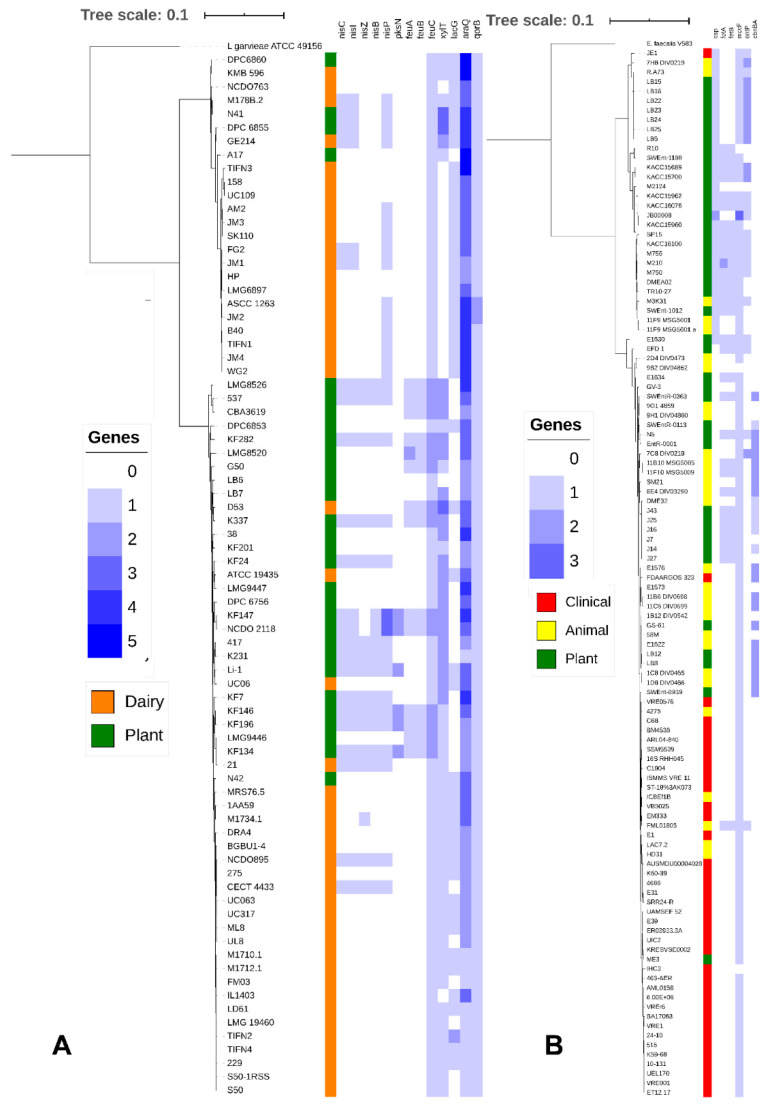
(**A**) Core genome tree of 77 *Lactococcus lactis* genomes. Genomes from *L. lactis* strains isolated from Dairy (Orange) and Plants (Green) are indicated, *Lactococcus garvieae* ATCC 49156 was included as an outgroup. A heatmap was generated indicating copy number of genes involved in nisin production (*nisC*, *nisI*, *nisZ*, *nisB*, and *nisP*), surfactin production (*PksN*), iron uptake (*feuA*, *feuB*, and *feuC*), xylose utilization (*xylT*), lactose utilization (*lacG*), arabinose utilization (*araQ*) and phenolic metabolism (*qorB*). (**B**) Core genome tree of 111 *Enterococcus faecium* genomes. Genomes from *E. faecium* strains isolated from clinical sources (Red), animals (Yellow), and plants (Green) are indicated, *Enterococcus faecalis* V583 is included as an outgroup. A heatmap was generated indicating the copy number of genes involved in cold shock response (*csp*), iron uptake (*fetA*, *fetB*), microcin (*mccF*), enterocin (*entP*), and carnobacteriocin (*cbnBA*).

To determine whether plant associated lactococci differ from those strains that are used in back-slopped dairy fermentations, comparative genomic analysis was performed on a total of 77 strains of *L. lactis*, isolated from two different ecosystems: two genomes of strains isolated in this study; 28 genomes, available on NCBI, of strains isolated from plants; and 47 genomes, available on NCBI, of strains isolated from either back-slopped dairy starter cultures or cheese produced with back-slopped cultures. The two lactococcal strains (LB6 and LB7) were randomly selected from the nine strains isolated in this study for genome sequencing. Likewise, comparative genomic analysis was performed on a total of 111 strains of *E. faecium*, isolated from different ecosystems: 9 genomes belonged to as many strains isolated in this study; the other 102 genomes were available on NCBI and, among these, 35 belonged to as many strains isolated from plant, soil or fresh water, 38 belonged to clinical isolates and 29 genomes to strains from healthy and non-domesticated animals. AntiSMASH identified genes coding for production of multiple antifungal lipopeptides in the genome of *B. velezensis* FUA 2155 and *B. amyloliquefaciens* Fad 82 (Table S2) but not in any of the genome of *L. lactis* and *E. faecium*. Core genome phylogenetic trees of *L. lactis* and *E. faecium* are shown in Figure 5.

Dairy isolates of *L. lactis* strains were dispersed in the tree but clustered in areas, representing L. *lactis* subsp. *cremoris* (upper cluster) and *L. lactis* subsp. *lactis* (lower cluster) (Figure 5A). Plant isolates of *L. lactis* were identified in both subspecies but predominantly belonged to *L. lactis* subsp. *lactis*. Over 2000 genes were differentially distributed in dairy and plant isolates of *L. lactis* (Benjamini-Hochberg adjusted *p* < 0.05) (Table S3). A majority of these genes were hypothetical proteins without known function, followed by genes coding for carbohydrate metabolism. Genes enriched in dairy isolates of *L. lactis* included a plasmid-encoded lactose PTS system, genes of the tagatose pathway for galactose utilization, genes coding for components of the oligopeptide transporter Opp, and genes coding for the manganese transporter MtnH and cystathionine-γ-lyase (Table S3 and Figure 5A). Genes that were enriched in plant associated lactococci include genes encoding for nisin biosynthesis (*nisC*, *nisI*, *nisZ*, *nisB*, and *nisP*), disaccharide and pentose metabolism (e.g., *xylT* and *araQ*), cystathionine-β-lyase, agmatine deiminase, iron uptake systems (*feuA*, *feuB*, and *feuC*), genes related to synthesis of the sideropore enterobactin, oleate hydratase, the quaternary ammonia compound resistance gene *qacC,* the lactose permease *lacF*, and the quinone oxidoreductase *qorB* (Table S3 and Figure 5A).

Clinical isolates of *E. faecium* were concentrated to a single phylogenetic clade (Figure 5B) although few clinical isolates also clustered in other clades. Plant isolates and isolates from wild animals that have little or no exposure to humans or antibiotics were represented on the tree without clear separation (Figure 5B). Strains of enterococci isolated from wheat rhizosphere predominantly, but not exclusively, clustered together (Figure 5B), likely reflecting that most but not all of these isolates were obtained from the same field (Table 1). *E. faecium* LB8 and LB12, isolated in this study, fell in the same phylogenetic clade as the clinical isolates. When comparing clinical and plant isolates of *E. faecium*, the gene content of 681 genes was differentially distributed (Benjamini-Hochberg adjusted *p* < 0.05) (Table S4); 606 genes were differentially distributed between clinical and animal isolates but only 400 genes were differentially distributed between plant and animal isolates (Table S4). Genes that were differentially distributed between clinical and plant isolates predominantly related to carbohydrate transport and metabolism but also included over 50 IS-transposases (Table S4 and Figure 5B). Genes that were enriched in plant associated enterococci also included genes coding for a cold shock protein (*csp*), the iron transport system *FetB*, and genes related to the synthesis of the bacteriocins microcin (*mccF*), enterocin P (*entB*), and carnobacteriocin A (*cbnBA*) (Table S4 and Figure 5B).

## 4. Discussion

Only few studies elucidated the role of lactic acid bacteria (LAB) in the rhizosphere and their plant growth promoting properties [18,19]. In our study, LAB were isolated from rhizospheric soil collected from two different cultivars of durum wheat during different growth stages. *L. lactis* subsp. *lactis* and *E. faecium* were the only two species of LAB found. Studies on microbial communities associated with roots showed that the growth stages [49,50,51], genotype [49,50], and soil type [51] influence their diversity. Among these factors, soil type is the main force driving changes in the community, followed by growth stage [51]. LAB represent only a small part of the wheat rhizosphere microbial community [52,53], therefore, enrichment methods are necessary to isolate them from soil [18]. In addition, LAB are fastidious microorganisms and are more easily detected in soils cultivated with fruit trees or, more in general, soils rich in carbon sources [54]. However, carbon sources from root exudates may also support growth of LAB in rhizospheric soil [55].

Research on *L. lactis* and *E. faecium* focused mainly on their role as dairy starter cultures and as nosocomial pathogens, respectively. *L. lactis* subsp. *lactis* and *L. lactis* subsp. *cremoris* are major components of mesophilic dairy starter cultures, which are maintained by back-slopping [56] but the organism is thought to originate from plant-associated habitats. The adaptation of *L. lactis* to the dairy environment relates to the acquisition of lactose metabolism, proteolysis and peptide transport; these genes are often encoded on plasmids [57,58]. Our comparative genomic analyses confirm these prior observations; the availability of a large number of genomes of lactococci of plant origin additionally allowed to identify genes that are enriched in plant-associated strains. Dairy isolates clustered in two distinct phylogenetic clades presenting L. *lactis* subsp. *cremoris* and *L. lactis* subsp. *lactis*; the close relationship of dairy isolates may reflect the fact that dairy cultures are descendants of only few strains that were isolated in the U.S. and Denmark [59]. Plant-associated strains were enriched in genes coding for disaccharide and pentose metabolism and were more likely to encode genes for synthesis of nisin variants. Plant strains also encoded for a polyketide synthase (PKS). The closest homologue to the PKS in *L. lactis* is the surfactin synthase in *B. subtilis* (GenBank accession no. Q04747). Because six out of 30 genomes of plant-associated strains also included a surfactin thioesterase, these strains are likely capable of producing the peptide antibiotic surfactin [60]. Plant-associated lactococci also encoded for agmatine deiminase (AgDI). Agmatine is the product of decarboxylation of arginine by *Enterobacteriaceae*; among Lactobacilli, AgDI is found only in the plant-associated genera *Levilactobacillus*, *Secundilactobacillus*, and *Lentilactobacillus* [61]. Plant tissue injury activates polyphenol oxidases and lipoxygenases which catalyse quinones and lipoperoxides as part of the plant stress response [62,63]. Plant-associated lactococci strains encoded a different ortholog of the quinone oxidoreductase QorB than dairy isolates. Moreover, seven plant strains encoded for oleate hydratase, which synthesizes antifungal oxylipins from linoleic acid [64].

*E. faecium* is usually associated with gastrointestinal tract of humans and animals [65] but is also relevant as a nosocomial pathogen causing life-threatening systemic infections. Most isolates from infected patients can be assigned to a single phylogenetic clade [23,66]. These observations were confirmed in our analyses, which also confirmed that mobile genetic elements, particularly transposases, strongly contribute to the diversification of plant strains, animal commensals, and human clinical isolates. Comparable to lactococci, genes coding for bacteriocin production were more frequent in plant-associated enterococci, indicating that antimicrobial activity against closely related strains [67] contributes to ecological fitness in plant ecosystems. Plant growth promotion properties were previously documented for a soil isolate of *E. faecium*, which secreted gibberellins and 3-indolacetic acid (IAA), thus improving the length and biomass of rice shoots [68].

That *E. faecium* LB8 and LB12 were closely related to clinical isolates of the same species and were thus not considered for further characterization. Investigating in vitro the properties of the LAB isolated from wheat rhizospheric soil was the first step to understand their potential plant growth promoting activity. We tested the capacity by LAB to produce IAA, because it is one of the main PGP traits of bacteria inhabiting rhizosphere [13]. No isolate produced IAA in physiologically relevant concentrations, in agreement with previous studies which also included also LAB [69,70]. On the other hand, Mussa et al. [71] reported that one strain of *Enterococcus casseliflavus*, isolated from grass pea rhizospheric soil, synthesized IAA at a concentration of ca. 56 µg mL^−1^, corresponding to about one order of magnitude higher than the best IAA producer among the LAB subjected to this research. Many rhizobacteria, including *Pseudomonas* sp. [72] *Bacillus* sp. [73] and, especially *Enterobacter* sp. [74,75,76] synthesize this phyto-hormone at concentrations of more than 100 µg·mL^−1^.

Phosphorus (P) is an important macronutrient for plant growth, but most of the soil phosphorus is not available for plants because it is either immobilized in soil in organic forms such as inositol phosphates or phosphonates or precipitated in inorganic form including apatites or Ca, Fe, and Al phosphates [77]. Even phosphorus applied as external input is quite often transformed into one of the unavailable forms [78]. Soil microorganisms play an important role in the mineralization and solubilization of organic and inorganic P respectively [79]. Under our experimental conditions, all the 16 LAB strains solubilized high amount of phosphate, comparable to strains of *Enterococcus* sp. isolated from grass pea rhizospheric soil [71]. The release of organic acids from LAB and the consequent acidification of broth (4.5 < pH < 4.9) could explain the observed solubilization of P. Indeed, previous researches showed inverse correlation between phosphate solubilization and pH of culture broth, varying from weak–moderate [71] to strong [80]. On the other hand, de Lacerda et al. [81] reported no significant correlation between pH and calcium phosphate solubilization. The same authors suggested that the ability of *L. lactis* to solubilize different sources of organic and inorganic P could be related to gene sequences possibly coding for two types of alkaline phosphatase, enzymes that catalyze the mineralization of phosphates [81].

Potassium (K) is another essential element for plants and its deficiency has a negative impact on roots, seeds, growth, and yields [82]. Although its concentration in soil solution is higher than P (generally 2–5 mg·L^−1^), most of K is in fixed forms [83]. Two potassium-solubilizing rhizobacteria (KSR), isolated from K-bearing minerals, and belonging to *Bacillus* sp., were proposed as an alternative to chemical K-containing fertilizers [82]. As far as we know, first our work showed LAB ability to solubilize K from mica powder. Strains of *E. faecium* proved to be better K-solubilizers than *L. lactis* subsp. *lactis*. The K-solubilizing activity of LAB could be due to formation of lactic acid and the corresponding acidification.

Defending plants from soil-borne pathogens is another feature of many rhizobacteria, being *Pseudomonas* and *Bacillus* currently the genera most used as biocontrol agents [84]. The antibacterial and antifungal activity of LAB is exploited in food production [67,85] but their use as biocontrol agents in crops is limited to in vitro studies [86] and to very few in situ applications. Cell-free supernatant of *Levilactobacillus brevis* JJ2P inhibited *Zymoseptoria tritici* in wheat seedling test, reducing the development of wheat leaf blotch [87]. Consortia of lactobacilli were inoculated in corn kernels, together with *Fusarium verticilloides*, a mycotoxigenic fungus causing ear rot. Compared to corn plants inoculated just with the phytopathogenic fungus, lactobacilli decreased disease severity and increased yield performance and the seed weight [88]. In a greenhouse experiment, a strain of *L. lactis* subsp. *lactis* was applied to foliar branches of holy basil inoculated with conidia of *Alternaria* sp. After 60 days post inoculation, the percentage of leaf blight in plants inoculated with the lactococcal strain was lower than the plants inoculated just with *Alternaria* sp. In field conditions, where basil plants had not been deliberately inoculated with *Alternaria* sp., plants inoculated with *Lc. lactis* subsp. *lactis* also showed a lower disease incidence than untreated control plants [89]. In the current study, three strains of lactococci and one *E. faecium* strain inhibited in vitro growth of the phytopathogenic fungus *F. graminearum* to an extent that was comparable to the two *Bacillus* sp. strains that were used as positive controls. However, the antifungal activity depended on the culture medium used for LAB. We may hypothesize that the four LAB strains synthesized cyclic dipeptides at concentrations higher than the MIC during growth on MRS [85], but not during growth on GYP. The two *Bacillus* strains displayed potent antifungal activity also against *P. roqueforti* and *A. niger*, and both strains harboured genes encoding for synthesis of several antifungal lipopeptides [39]. Remarkably, genes related to biosynthesis of surfactin in *Bacillus subtilis* were identified in six genomes of plant-associated lactococci. Surfactin does not display strong antifungal activity but was reported to act synergistically with other antifungal compounds [90] and thus may enhance the antifungal activity of bacilli.

Food preservation with LAB relies mainly on the acidification; bacteriocins specifically enhance the inhibitory activity of LAB towards Gram-positive bacteria, but bacteriocins of LAB are generally inactive against Gram-negative bacteria [67,91]. Comparative genomic analyses presented in this study identified bacteriocin production as a part of the plant-associated lifestyle of lactococci and enterococci but also documented only weak and pH-dependent antibacterial activity with Gram-negative phytopathogens as indicator strains. Lactic acid production, however, permeabilizes the outer membrane of Gram-negative bacteria and increases their sensitivity to hydrophobic inhibitors [92]. Lactic acid production in association with the production of other antibacterial compounds may account for the protective effect of a LAB consortium against papaya infection with *Erwinia mallotivora* [93].

## 5. Conclusions

This study showed that, besides the well-recognized PGPB, LAB isolated from wheat rhizosphere may have potential plant growth promotion activities, too. No strain possessed all the plant growth promoting activity at the highest level and therefore ongoing research is focusing on setting-up a LAB consortium for future application as biofertilizer in conventional and organic agriculture. LAB showed only weak inhibitory activity against plant pathogenic fungi and Gram-negative bacteria, but may synergistically enhance the activity of compounds produced by other members of the rhizosphere. Plant growth promotion and efficacy of biocontrol should obviously be confirmed under outdoor conditions, where cultivar and soil type, among others, could affect plant response [94]. Comparative genomics also allowed to differentiate LAB with potential plant growth promotion and biocontrol activity from food-related strains of *L. lactis* and from strains of *E. faecium* isolated from humans.

## Figures and Tables

**Table 1:** Lactic acid bacteria isolated from wheat rhizopsheric soil collected from two different experimental sites during three different growth stages.

Geographic Origin	Growth Stage *	Medium of Isolation	Strain Name ^†^	Relative Abundance ^¥^	Closest Relative ^&^	Genome Accession Number
Gravina	Elongation	MRS	LB1	9.1%	*L. lactis*	
Gravina	Elongation	MRS	LB2	11%	*L. lactis*	
Gravina	Elongation	MRS	LB3	5.5%	*L. lactis*	
Gravina	Elongation	MRS	LB4	18.2%	*L. lactis*	
Turi	Elongation	MRS	LB7	14.5%	*L. lactis*	JADBCD000000000
Turi	Elongation	MRS	LB8	5.4%	*E. faecium*	JADBCC000000000
Turi	Elongation	MRS	LB9	14.5%	*L. lactis*	
Turi	Elongation	MRS	LB10	11%	*L. lactis*	
Turi	Elongation	MRS	LB11	3.6%	*L. lactis*	
Turi	Elongation	MRS	LB12	7.2%	*E. faecium*	JADBCB000000000
Turi	Earing	MRS	LB5	15.5%	*E. faecium*	JADBCF000000000
Turi	Earing	MRS	LB6	32.4%	*L. lactis*	JADBCE000000000
Turi	Earing	GYP	LB15	24%	*E. faecium*	JADBCA000000000
Turi	Earing	GYP	LB16	28.1%	*E. faecium*	JADBBZ000000000
Turi	Physiological maturity	GYP	LB22	13.3%	*E. faecium*	JADBBY000000000
Turi	Physiological maturity	GYP	LB23	26.7%	*E. faecium*	JADBBX000000000
Gravina	Physiological maturity	GYP	LB24	6.7%	*E. faecium*	JADBBW000000000
Turi	Physiological maturity	GYP	LB25	13.3%	*E. faecium*	JADBBV000000000

* The 10 bacterial strains isolated during tillering stage have identical RAPD patterns as other strains and were not further identified. ^†^ LAB were biotyped upon RAPD-PCR. ^¥^ Relative abundance of biotypes was calculated as the percentage ratio between the number of bacterial isolates belonging to a given biotype and the total number of bacterial isolates in a given growth stage. ^&^ all *L. lactis* strains matched to *L. lactis* subspecies *lactis*. The nucleotide identity of all sequences to the closest relative was greater than 99% for all strains.

**Table 2:** Values of phosphate solubilization index measured for LAB strains isolated from wheat rhizospheric soil.

LAB STRAIN	Phosphate Solubilization Index (mm) *
*L. lactis* subsp. *lactis* LB1	1.69 ± 0.13 ^b–e^
*L. lactis* subsp. *lactis* LB2	1.65 ± 0.07 ^c–f^
*L. lactis* subsp. *lactis* LB3	1.60 ± 0.05 ^d–g^
*L. lactis* subsp. *lactis* LB4	1.75 ± 0.15 ^a–d^
*E. faecium* LB5	1.02 ± 0.03 ^i^
*L. lactis* subsp. *lactis* LB6	1.17 ± 0.17 ^h,i^
*L. lactis* subsp. *lactis* LB7	1.11 ± 0.05 ^h,i^
*L. lactis* subsp. *lactis* LB9	1.46 ± 0.04 ^g^
*L. lactis* subsp. *lactis* LB10	1.87 ± 0.11 ^a^
*L. lactis* subsp. *lactis* LB11	1.67 ± 0.00 ^b–e^
*E. faecium* LB15	1.78 ± 0.1 ^a–c^
*E. faecium* LB16	1.83 ± 0.17 ^a,b^
*E. faecium* LB22	1.66 ± 0.13 ^b–f^
*E. faecium* LB23	1.55 ± 0.08 ^e–g^
*E. faecium* LB24	1.22 ± 0.1 ^h^
*E. faecium* LB25	1.50 ± 0.0 ^f,g^

* Values are the mean (±SD) of three replicates. Values with no common letters (^a–i^) indicate significant differences at 5% probability level (LSD test).

**Table 3:** Values of potassium solubilization index measured for LAB strains isolated from wheat rhizospheric soil and corresponding classification.

LAB STRAIN	Potassium Solubilization Index (mm) *	Classification **
*Lactococcus lactis* subsp. *lactis* LB1	1.9 ± 0.1 ^c–e^	++
*L. lactis* subsp. *lactis* LB2	1.9 ± 0.1 ^c–e^	++
*L. lactis* subsp. *lactis* LB3	2.0 ± 0.3 ^c,d^	++
*L. lactis* subsp. *lactis* LB4	2.0 ± 0.1 ^c,d^	++
*Enterococcus faecium* LB5	0.0 ± 0.0 ^f^	−
*L. lactis* subsp. *lactis* LB6	0.0 ± 0.0 ^f^	−
*L. lactis* subsp. *lactis* LB7	0.0 ± 0.0 ^f^	−
*L. lactis* subsp. *lactis* LB9	1.7 ± 0.1 ^e^	++
*L. lactis* subsp. *lactis* LB10	1.7 ± 0.1 ^d,e^	++
*L. lactis* subsp. *lactis* LB11	1.8 ± 0.1 ^d,e^	++
*E. faecium* LB15	2.5 ± 0.5 ^a^	+++
*E. faecium* LB16	2.1 ± 0.3 ^b,c^	+++
*E. faecium* LB22	0.0 ± 0.0 ^f^	−
*E. faecium* LB23	2.4 ± 0.1 ^a,b^	+++
*E. faecium* LB24	2.1 ± 0.1 ^c^	+++
*E. faecium* LB25	2.1 ± 0.2 ^c^	+++

* Values are the mean (±SD) of three replicates. Values with no common letters (^a–f^) indicate significant differences at 5% probability level (LSD test). ** −, no halo zone; ++, 1.5 mm ≤ halo zone ≤ 2 mm; +++, > 2 mm halo zone.

## Data Availability

Data is contained within the article or supplementary material.

## Supplementary Materials

The following are available online at https://www.mdpi.com/2076-2607/9/1/78/s1, Table S1: MIC (% vol/vol) values of the cell-free supernatant of *L. lactis* subsp. *lactis* strains against phytopathogenic bacteria; Table S2: Secondary metabolites produced by *Bacillus* spp; Table S3: genes’ distribution between dairy and plant isolates of *L. lactis*; Table S4: genes’ distribution between clinical and animal isolates of *E. faecium*.
