# Supplementary material for: Comparative Genomics and In Vitro Plant Growth Promotion and Biocontrol Traits of Lactic Acid Bacteria from the Wheat Rhizosphere
Source: Microorganisms. 2020 Dec 30;9(1):78. doi: 10.3390/microorganisms9010078 (PMC7823429; doi:10.3390/microorganisms9010078)
Supplement: Supplementary file 1 [file microorganisms-09-00078-s001.zip › microorganisms-1035878 proofed supplementary.docx]

Table S1. MIC (% vol/vol) values of the cell-free supernatant of *L. lactis* subsp. *lactis* strains against phytopathogenic bacteria.

| ***L. lactis* subsp. *lactis*** **strain** | ***Pseudomonas syringae*** | ***Pectobacterium rhapontici*** | ***Pantoea agglomerans*** | ***Pectobacterium carotovorum*** | ***Pseudomonas fulva*** |
| --- | --- | --- | --- | --- | --- |
| LB1 | 2.1^b^ | 4.2^a^ | 2.1^a^ | 4.2^a^ | 8.3^b^ |
| LB2 | 2.1^b^ | 4.2^a^ | 2.1^a^ | 4.2^a^ | 8.3^b^ |
| LB3 | 2.1^b^ | 4.2^a^ | 2.1^a^ | 4.2^a^ | 8.3^b^ |
| LB4 | 4.2^a^ | 4.2^a^ | 2.1^a^ | 4.2^a^ | 8.3^b^ |
| LB6 | NA^*c^ | NA^b^ | NA^b^ | NA^c^ | NA^c^ |
| LB7 | NA^c^ | NA^b^ | NA^b^ | NA^c^ | NA^c^ |
| LB9 | 4.2^a^ | 4.2^a^ | 2.1^a^ | 4.2^a^ | 16.7^a^ |
| LB10 | 2.1^b^ | 4.2^a^ | 2.1^a^ | 4.2^a^ | 8.3^b^ |
| LB11 | 2.1^b^ | 4.2^a^ | 2.1^a^ | 2.1^b^ | 16.7^a^ |

Values are the mean of three replicates. Values in the same column with no common letters (a–c) indicate significant differences at 5% probability level (LSD test).

^*^ NA, no activity against phytopathogenic bacteria.

**Table S2.** Secondary metabolites produced by *Bacillus* spp.

| ***Bacillus* species** | **Secondary metabolites** |
| --- | --- |
| ***B. velezensis* FUA 2155** | Macrolactin, Bacillaene, Fengycin, Bacillibactin, Bacilysin, Difficidin |
| ***B. amyloliquefaciens* Fad 82** | Bacillaene, Bacilysin, Bacillibactin |

The production of secondary metabolites was predicted using the genome sequences of *B. velezensis* FUA 2155 (GenBank Accession number SDKI00000000) and *B. amyloliquefaciens* Fad 82 (GenBank Accession number SDKG00000000) [38] and antiSMASH 5.0 [95].

38. Li, Z.; Schottroff, F.; Simpson, D.J.; Gänzle, M.G. The copy number of the *spoVA*^2mob^ operon determines pressure resistance of *Bacillus* endospores. *Appl. Environ. Microbiol.* **2019,** 85, e01596-19. https://doi.org/10.1128/aem.01596-19.

95. Blin, K.; Shaw, S.; Steinke, K.; Villebro, R.; Ziemert, N.; Lee, S.Y.; Medema, M.H.; Weber, T. AntiSMASH 5.0: Updates to the secondary metabolite genome mining pipeline. *Nucleic Acids Res.* **2019,** 47, W81–W87. https://doi.org/10.1093/nar/gkz310.
